# Longitudinal Measurement of Histidine-Rich Glycoprotein Levels in Bronchopulmonary Dysplasia: A Pilot Study

**DOI:** 10.3390/biomedicines11010212

**Published:** 2023-01-14

**Authors:** Daisaku Morimoto, Yosuke Washio, Kei Tamai, Takeshi Sato, Tomoka Okamura, Hirokazu Watanabe, Yu Fukushima, Junko Yoshimoto, Misao Kageyama, Kenji Baba, Hirokazu Tsukahara

**Affiliations:** 1 Department of Pediatrics, Graduate School of Medicine, Dentistry and Pharmaceutical Sciences, Okayama University, 2-5-1 Shikatacho Kita-ku, Okayama 700-8558, Japan; 2 Division of Neonatology, Okayama Medical Center, National Hospital Organization, Okayama, 1711-1 Tamasu Kita-ku, Okayama 701-1192, Japan

**Keywords:** extremely preterm infants, histidine-rich glycoprotein, high mobility group box 1, bronchopulmonary dysplasia, longitudinal measurement, mixed-effects model

## Abstract

Histidine-rich glycoprotein (HRG) has been reported to inhibit signaling leading to the release of high mobility group box 1 protein, a damage-associated molecular pattern. The present study aimed to determine the longitudinal change in HRG levels in extremely preterm infants and assess whether complications such as bronchopulmonary dysplasia (BPD) were associated with differences in HRG levels. In this multicenter, prospective, observational study, we measured serum HRG levels every 2 weeks from birth to 8 weeks of age. Serum HRG was measured using an enzyme-linked immunosorbent assay. We included 19 extremely preterm infants in the study and 74 samples were analyzed. The median gestational age was 26.0 weeks, and the median birth weight was 858 g. Serum HRG levels showed a significant upward trend after birth (*p* < 0.001); median HRG concentrations at birth and at 2, 4, 6, and 8 weeks of age were 1.07, 1.11, 2.86, 6.05, and 7.49 µg/mL, respectively. Onset of BPD was not associated with differences in serum HRG levels. Further, the serum HRG levels increased significantly after birth in extremely preterm infants.

## 1. Introduction

Histidine-rich glycoprotein (HRG) is a plasma protein with a molecular weight of 75 kDa, synthesized in the liver, monocytes, macrophages, and megakaryocytes [1,2,3,4]. HRG has various physiological effects, including the modulation of immune complex formation, antibacterial and antifungal effects, modulation of angiogenesis, and antithrombotic effects [5,6,7,8].

HRG has been reported to inhibit signals leading to the release of high mobility group box 1 protein (HMGB-1) and damage-associated molecular patterns (DAMPs) linked to various pathologies, including sepsis [9,10]. HMGB-1 levels are persistently elevated in bronchopulmonary dysplasia (BPD), which affects the long-term prognosis of extremely preterm infants, and HRG may be indirectly involved in the pathogenesis of BPD in extremely preterm infants [11].

Although preterm infants have been reported to have lower HRG values at birth than adults, no studies have measured subsequent trends over time [12]. We hypothesized that postnatal HRG values would be associated with subsequent comorbidities such as BPD.

The purpose of this study was to determine the longitudinal change in HRG in extremely preterm infants and evaluate whether comorbidities such as BPD were associated with differences in HRG. Identifying changes in the HRG of infants with comorbidities may lead to the development of new treatments, such as HRG-rich fresh frozen plasma, for diseases in extremely preterm infants.

## 2. Materials and Methods

### 2.1. Patient Selection

This prospective, observational study was planned at Okayama University (Okayama, Japan) and was conducted with the cooperation of the Okayama Medical Center (Okayama, Japan). We included infants born at less than 28 weeks of gestation and admitted to either institution between April 2016 and March 2022. Written informed consent was obtained from the parents of the patients. Infants with chromosomal abnormalities, complex cardiac malformations, central nervous system abnormalities, inborn errors of metabolism, or immunodeficiency syndromes, as well as any infants deemed unsuitable for participation by the attending physicians, were excluded.

### 2.2. Definition of Comorbidities

Comorbidities were defined as follows. Respiratory distress syndrome (RDS) was diagnosed using a stable microbubble rating and chest radiograph before surfactant replacement [13]. BPD was diagnosed based on an abnormal chest radiograph at 28 days of age and the need for oxygen support. BPD36 was defined as moderate or severe BPD at 36 postmenstrual weeks [14]. Hemodynamically significant patent ductus arteriosus and intraventricular hemorrhage were diagnosed by clinical symptoms and ultrasonography [15]. Sepsis was diagnosed by blood culture tests. A diagnosis of necrotizing enterocolitis was made based on blood tests and an abdominal radiograph [16]. Clinical chorioamnionitis was diagnosed based on Lencki’s criteria [17]. Retinopathy of prematurity (ROP) was diagnosed based on fundus examination and treated when it reached stage 2 or higher according to the International Classification of ROP [18].

### 2.3. Data Collection

Using electronic medical records, we obtained the following data: gestational age, birth weight, sex, presence or absence of the aforementioned complications, gravidity, parity, cesarean section, Apgar score, umbilical cord artery blood pH and its base excess.

Specimens were collected from the umbilical venous blood at birth and peripheral venous blood every 2 weeks until 36 corrected weeks (maximum 8 weeks old). Sample collection was delayed for up to 1 week, depending on the infant’s condition. After the specimens were collected, the serum was centrifuged and stored at −20 °C. HRG was measured using an enzyme-linked immunosorbent assay kit (Human HRG ELISA Kit, Abcam, UK).

### 2.4. Endpoints and Statistical Analysis

The primary endpoint was the difference in HRG between infants with and without comorbidities such as BPD36. The secondary endpoint was serum HRG concentration at each time point.

For statistical analysis, the Mann–Whitney U test and Fisher’s exact test were used to compare baseline characteristics. Values are presented as number (percentage) or median (interquartile range). Comparison of HRG values with and without comorbidities was performed by repeated measures analysis of variance using a mixed-effects model. Statistical significance was set at *p* < 0.05. The statistical software used were R 4.1.1 (R Foundation for Statistical Computing, Vienna, Austria) and the lmerTest package.

### 2.5. Ethical Aspects

This study was approved by the Ethics Committee of Okayama University (No. K1902-036 approved on 1 March 2019 and No. K1510-016 approved on 27 October 2015).

## 3. Results

### 3.1. Baseline Characteristics of the Patients

This study included 19 extremely preterm infants with parental consent. Blood samples were collected from the cord blood at birth to 8 weeks of age, and 74 samples were analyzed. The median gestational age was 26.0 weeks, and the median birth weight was 858 g. Table 1 shows the baseline characteristics of infants.

### 3.2. Comparisons by Comorbidities

Comparisons of the primary endpoint are shown in Table 2. We compared 8 cases in the BPD36 group with 10 cases in the non-BPD group; one case was excluded due to missing endpoint records. The BPD36 group had a significantly shorter gestational age. Birthweight and Apgar score at 1 min also had a tendency to be lower in the BPD36 group.

Table 3 shows the results of the analysis of variance comparing serum HRG longitudinal measurements with and without complications. Using a mixed-effects model, differences in HRG were analyzed for weeks of age and postmenstrual weeks, respectively. This additional analysis was performed due to the shorter gestational age in the BPD36 group. In both analyses, there was no difference in the distribution of HRG values for all complications.

### 3.3. Longitudinal Change in HRG Levels in All Infants

Serum HRG levels showed a statistically significant upward trend after birth (*p* < 0.001) (Table 4). Post-hoc analysis showed significant differences between cord blood and blood collected at 8 weeks old (*p* = 0.023); blood collected at 2 weeks old and 6 weeks old (*p* = 0.049); blood collected at 2 weeks old and 8 weeks old (*p* = 0.044).

## 4. Discussion

This is the first study to measure the postnatal course of serum HRG levels in extremely preterm infants. The HRG concentration at birth was 1.07 μg/mL, which was lower than the concentrations reported in term infants and adults in previous studies (12–16 μg/mL and 86 μg/mL, respectively) [12,19]. Additionally, to the best of our knowledge, the present study is the first to reveal a slow increase in HRG until the near-term period.

HRG was discovered by Heimburger et al. in 1972 [1]. In 1990, Corrigan et al. and Morgan et al. reported HRG concentrations in healthy neonates [12,19]. However, that of extremely preterm infants and its effect on comorbidities such as BPD were not well known.

Recently, HRG has been reported to be associated with HMGB-1 and DAMPs. Gao et al. demonstrated that HRG suppressed HMGB-1-induced inflammatory responses and identified the associated HRG receptors [10]. HMGB-1 has been reported to be associated with the development of BPD in extremely preterm infants [11]. In a mouse model, it was reported that administration of anti-HMGB-1 antibody prevented exacerbation of BPD [20]. Thus, HRG may prevent BPD and other complications in preterm infants by preventing the HMGB-1 pathway.

This study may be one of the first to clarify the connection between comorbidities in extremely preterm infants and HRG. The association between shorter postmenstrual weeks and lower HRG levels led to the expectation that the suppression of the activity of HMGB-1, a DAMP, would be less effective in extremely premature infants. However, in the present study, there was no clear difference in HRG values based on the presence or absence of comorbidities. We speculated on two reasons for these negative results. First, the number of study participants in the present study was small; there was a trend toward slightly lower HRG levels in the BPD group, which could be significant with a larger number of study participants. Second, premature infants who develop BPD and/or other comorbidities are often given blood products, including fresh frozen plasma. Because HRG is contained in these products, the administration of such products may affect the HRG level of the infant.

To compensate for the small sample size in the present study, we used a mixed-effects model [21]. While traditional repeated measures analysis of variance requires the exclusion of cases with missing values, this model allows us to include cases with missing values. However, this analysis also failed to demonstrate significant results.

In conclusion, serum HRG levels were not different between BPD infants and non-BPD infants. Serum HRG levels significantly increased after birth in extremely preterm infants. Additional research and analysis of more cases might reveal differences in HRG due to comorbidities.

## Figures and Tables

**Table 1:** Characteristics of included infants.

	All Infants (*n* = 19)
Male sex; *n* (%)	10 (53%)
Gestational age; week (w) and day (d)	26 w 0 d (25 w 0 d–27 w 0 d)
Birthweight; g	858 (618–981)
Primipara; *n* (%)	6 (32%)
Cesarean section; *n* (%)	10 (53%)
Apgar score at 1 min	4 (2.5–5)
Apgar score at 5 min	7 (6–7)
UA pH	7.320 (7.269–7.387)
UA base excess	−3.6 (−8.1 to −0.9)
RDS; *n* (%)	11 (58%)
BPD36; *n* (%)	8/18 (44%) *
HsPDA; *n* (%)	11 (58%)
IVH; *n* (%)	8 (42%)
Sepsis; *n* (%)	5 (26%)
NEC; *n* (%)	2 (11%)
Clinical CAM; *n* (%)	4 (21%)
ROP treatment; *n* (%)	3 (16%)

Values are presented as number (percentage) or median (interquartile range). UA: umbilical artery, RDS: respiratory distress syndrome, BPD36: bronchopulmonary dysplasia at 36 postmenstrual weeks, HsPDA: hemodynamically significant patent ductus arteriosus, IVH: intraventricular hemorrhage, NEC: necrotizing enterocolitis, CAM: chorioamnionitis, ROP: retinopathy of prematurity. * missing value in one case.

**Table 2:** Comparisons of Characteristics of BPD36 and non-BPD36 infants.

	BPD36 (*n* = 8)	Non-BPD36 (*n* = 10)	*p* Value
Male sex; *n* (%)	4 (50%)	5 (50%)	1
Gestational age; week (w) and day (d)	24 w 6 d (24 w 0 d–26 w 0 d)	27 w 0 d (25 w 2 d–27 w 0 d)	0.039 *
Birthweight; g	618 (541–803)	933 (841–1012)	0.055 ^†^
Primipara, *n*	4 (50%)	1 (10%)	0.118
Cesarean section; *n* (%)	4 (50%)	6 (60%)	1
Apgar score at 1 min	3 (2–4)	5 (4–6)	0.056 †
Apgar score at 5 min	6 (5–7)	7 (7–7)	0.167
UA pH	7.330 (7.248–7.364)	7.330 (7.274–7.404)	0.733
UA base excess	−4.1 (−8.9 to −0.5)	−3.1 (−8.1 to −1.2)	0.922
RDS; *n* (%)	5 (%)	5 (50%)	0.664
BPD; *n* (%)	8 (100%)	8 (80%)	0.477
HsPDA; *n* (%)	3 (%)	7 (70%)	0.342
IVH; *n* (%)	4 (50%)	3 (30%)	0.631
Sepsis; *n* (%)	3 (%)	1 (10%)	0.275
NEC; *n* (%)	1 (%)	1 (10%)	1
Clinical CAM; *n* (%)	2 (%)	2 (20%)	1
ROP treatment; *n* (%)	1 (%)	2 (20%)	1

Values are presented as number (percentage) or median (interquartile range). Continuous variables were analyzed using the Mann–Whitney U test, and categorical variables were analyzed using Fisher’s exact test. UA: umbilical artery, RDS: respiratory distress syndrome, BPD36: bronchopulmonary dysplasia at 36 postmenstrual weeks, HsPDA: hemodynamically significant patent ductus arteriosus, IVH: intraventricular hemorrhage, NEC: necrotizing enterocolitis, CAM: chorioamnionitis, ROP: retinopathy of prematurity. * *p* < 0.05; ^†^ *p* < 0.10.

**Table 3:** Comparison of HRG Longitudinal Measurements with and without Complications.

	Comparison in Weeks of Age	Comparison in Postmenstrual Week
BPD36	0.55	0.45
RDS	0.73	0.40
HsPDA	0.79	0.57
IVH	0.29	0.06
Sepsis	0.26	0.24
NEC	0.24	0.58
Clinical CAM	0.81	0.66
ROP treatment	0.28	0.15

Values are presented as *p*-values. Mixed-effects models were used to analyze comorbidities and time as fixed effects and cases as random effects. HRG: histidine-rich glycoprotein, BPD36: bronchopulmonary dysplasia at 36 postmenstrual weeks, RDS: respiratory distress syndrome, HsPDA: hemodynamically significant patent ductus arteriosus, IVH: intraventricular hemorrhage, NEC: necrotizing enterocolitis, CAM: chorioamnionitis, ROP: retinopathy of prematurity.

**Table 4:** Longitudinal Change in HRG.

Time Point	HRG (µg/mL)
Umbilical venous blood	1.07 (0.79–1.57)
Week 2	1.11 (0.73–1.91)
Week 4	2.86 (1.83–4.48)
Week 6	6.05 (4.08–8.52)
Week 8	7.49 (5.81–8.78)

Values are presented as median (interquartile range). HRG: histidine-rich glycoprotein.

## Data Availability

The data presented in this study are available on request from the corresponding author. The data are not publicly available due to privacy reasons.
